# Genital Infection with Herpes Simplex Virus Types 1 and 2 in Women from Natal, Brazil

**DOI:** 10.1155/2014/323657

**Published:** 2014-03-11

**Authors:** Cleine Aglacy Nunes Miranda, Érika Galvão Lima, Diego Breno Soares de Lima, Ricardo Ney Oliveira Cobucci, Maria da Conceição de Mesquita Cornetta, Thales Allyrio Araújo de Medeiros Fernandes, Paulo Roberto Medeiros de Azevedo, Jenner Chrystian Veríssimo de Azevedo, Josélio Maria Galvão de Araújo, José Veríssimo Fernandes

**Affiliations:** ^1^Post-Graduate Program in Biological Sciences, Federal University of Rio Grande do Norte, Avenida Salgado Filho, S/N, Campus Universitario, Lagoa Nova, 59072-970 Natal, RN, Brazil; ^2^Post-Graduate Program in Health Sciences, Federal University of Rio Grande do Norte, Avenida General Gustavo de Farias, S/N, Petropolis, 59012-570 Natal, RN, Brazil; ^3^Department of Tocogynecology, Federal University of Rio Grande do Norte, Avenida Nilo Peçanha 259, Petropolis, 59012-300 Natal, RN, Brazil; ^4^Department of Biomedical Sciences, University of Rio Grande do Norte State, Rua Miguel Antonio da Silva Neto, S/N, Aeroporto, 59607-360 Mossoro, RN, Brazil; ^5^Department of Statistics, Federal University of Rio Grande do Norte, Avenida Salgado Filho, S/N, Campus Universitario, Lagoa Nova, 59078-970 Natal, RN, Brazil; ^6^Pediatric Hospital, Federal University of Rio Grande do Norte, Avenida General Gustavo de Farias, S/N, Petropolis, 59012-570 Natal, RN, Brazil; ^7^Department of Microbiology and Parasitology, Federal University of Rio Grande do Norte, Avenida Salgado Filho, S/N, Campus Universitario, Lagoa Nova, 59072-970 Natal, RN, Brazil

## Abstract

*Objective*. To evaluate the prevalence of HSV-1 and HSV-2 in pregnant and nonpregnant women, testing the correlation between DNA of the viruses with colposcopic and/or cytological changes, and evaluate association with sociodemographic characteristics and sexual activity. *Methods*. Included in this study were 106 pregnant and 130 nonpregnant women treated at primary health care units of Natal, Brazil, in the period 2010-2011. The patients were examined by colposcopy, and two cervical specimens were collected: one for cytology examination and another for analysis by PCR for detection of HSV-1 and HSV-2. *Results*. HSV-1 alone was detected in 16.0% of pregnant and 30.0% of nonpregnant women. For HSV-2, these rates were 12.3% and 15.5%, respectively. HSV-2 had a higher correlation with cytology and/or colposcopy changes than HSV-1 did. Genital HSV-1 infection was not associated with any of the variables tested, whereas HSV-2 infection was associated with ethnicity, marital status, and number of sexual partners. *Conclusions*. The prevalence of HSV-1 was higher than that observed for HSV-2 in both pregnant and nonpregnant women. The genital infection by HSV-2 was higher in women with changed colposcopy and/or cytology, and it was associated with ethnicity, marital status, and number of sexual partners.

## 1. Introduction

Herpes simplex virus (HSV) is a neurotropic virus that has a large linear, double-stranded DNA genome protected by a capsid with icosahedral symmetry surrounded by an envelope consisting of a lipid bilayer with embedded glycoproteins, having yet a proteinaceous region between the capsid and envelope called tegument [1]. The HSV belongs to the family of Herpesviridae, subfamily Alphaherpesvirinae, and genus Simplex virus [2, 3]. It is a virus that has a very complex life cycle and stands out as one of the most common pathogens in the etiology of sexually transmitted diseases worldwide [4].

HSV infects the mucosa of the mouth, eyes, and the human anogenital tract. After primary infection, the virus replicates productively within mucosal epithelial cells and enters sensory neurons via nerve termini. The virus is then transported to neuronal cell bodies where latency is established. The virus can remain in this latent state indefinitely but can be reactivated at any time during the lifetime of the host [4, 5]. During latent infection, no infectious virus is produced from infected cells, symptoms are absent in the host, and the transmission does not occur. However, reactivation can occur only in some cells, in the absence of symptoms, enabling the transmission of the virus. This seemingly benign strategy has critical importance to the survival of the virus, where the infected cell continues throughout the life of the host, providing a reservoir for periodic reactivation [5].

HSV is presented in the form of two distinct serotypes: Herpes simplex type 1 (HSV-1) and type 2 (HSV-2), which are closely related but contain sufficient differences to enable type identification [5]. HSV-1 is usually transmitted during childhood by nonsexual contact, typically found in orofacial lesions, with latency in the trigeminal ganglia. HSV-2 is the cause of most genital herpes and is almost always sexually transmitted, with latency in the lumbosacral ganglia [6]. However, in the last decades, HSV-1 has been pointed out by several studies as a main causative agent of genital herpes, especially in college students [7, 8].

Regardless of which serotype of HSV is acquired, the infections remain intermittent throughout the host's lifetime, in symptomatic or subclinical form with or without periodic reactivations. The extent and frequency of recurrent genital herpes are highly type-specific, with HSV-2 reactivation outpacing HSV-1 by three to one. Thus, although an increase in genital infections attributable to HSV-1 has been well recognized in recent years, the situation regarding recurrence has apparently remained unaltered, with HSV-2 being the dominant agent [9]. When there is a reactivation, the virus is excreted on the mucosal surfaces, allowing its transmission to susceptible individuals. Most HSV-1 and HSV-2 infections are subclinical. However, when the infection is symptomatic, the clinical manifestations of HSV-2 are typically characterized by recurrent, painful, vesicular, and ulcerative lesions, located in the genital and anal areas. In contrast, symptomatic HSV-1 infections are usually manifested as recurrent orolabial and facial lesions [10].

Both HSV-1 and HSV-2 can cause genital herpes with greatest incidence among women of reproductive age, with risk of maternal transmission of the virus to the fetus and neonate [2]. The acquisition of genital herpes during pregnancy has been associated with spontaneous abortion, intrauterine growth restriction, prematurity, and congenital and neonatal herpes. Vertical transmission from an infected mother to her baby can cause severe disease resulting in sequelae or death of the infant. Most neonatal infections result from exposure to HSV in the genital tract during passage through the birth canal, although they can also be transmitted to the fetus during the intrauterine phase [11]. The risk of disease in the newborn is significantly higher when the mother acquires genital infection for the first time with HSV-1 or HSV-2 during pregnancy. Recurrent infections are rarely associated with disseminated neonatal disease in the newborn of immune-competent mothers. In fact, the pregnant women who acquire genital herpes as a primary infection in the latter half of pregnancy, rather than prior to pregnancy, are at the greatest risk of transmitting the virus to their newborn [2, 3].

Neonatal herpes has three major categories: skin, eye, and mouth disease (SEM), central nervous system infection (CNS), and disseminated infection (DI). It has been observed that SEM in itself is rarely fatal, but, without antiviral therapy, most cases progress to CNS or DI. Clinically, CNS disease typically presents with nonspecific symptoms such as lethargy, poor feeding, irritability, vomiting, and seizures. The disseminated infection is manifested in various combinations such as hepatitis, acute adrenal insufficiency, myocarditis, and consumption coagulopathy [12].

In Brazil, there is a major lack of studies on the prevalence of genital infection by herpes simplex virus. Furthermore, the diagnosis of genital infection with these pathogens is not included among the mandatory prenatal examinations. Thus, there is no official data available on infection during pregnancy nor on the likely consequences of such infections in newborns. In this study we evaluated the prevalence for herpes simplex virus types 1 and 2 genital infection among pregnant and nonpregnant women, its association with the presence of cervical abnormalities detected by colposcopy and cytology, sociodemographic characteristics, and reproductive activity.

## 2. Material and Methods

### 2.1. Selection of Participants

This study included 236 sexually active women, 106 (44.9%) of whom were pregnant and 130 (55.1%) nonpregnant, enrolled among those who attended the health service by spontaneous demand for cervical screening programme or to prenatal care, and agreed to participate in the study. The participants were residents in Natal, Rio Grande do Norte State, Brazil, who sought gynecological care at two primary health care units from May 2010 until September 2011 and who agreed to participate in the research. No patient had clinical signs of herpetic infection.

Study subjects were asked to fill in an anonymous questionnaire in order to identify different demographic and behavioral risk factors that may have implied their exposure to HSV-1 and HSV-2. The patient's ethnicity was identified based on self-reports according to the criterion of the Brazilian Institute of Geography and Statistics (IBGE), which classifies ethnicity into five categories: white, black, mulatto, Asian, and native. In this study, the black, mulatto, Asian, and native categories were combined into a nonwhite category. Demographic data included the age, level of education, and marital status. Data on sexual behavior included the age at the first sexual intercourse and the number of sexual partners during the lifetime.

The study was approved by the Ethics Committee for Medical Research of the Federal University of Rio Grande do Norte (Protocol 030/10—CEP/UFRN) and the written informed consent has been obtained from each subject.

### 2.2. Colposcopy and Cytopathological Analysis

All women participating in this study underwent a clinical examination followed by a visual inspection of the vulva, vaginal walls, and cervix by colposcopy, with only two gynecologists, to detect possible abnormalities of these structures. During the examination, a solution of 5% acetic acid was applied initially and the first visual analysis was performed. After washing to remove the acetic acid, lugol solution of I_2_ (1%) in equilibrium with KI (2%) in distilled water was applied, and the second inspection was done. The colposcopy was considered with alteration, when at least one of the abnormal features (acetowhite lesions, punctuation, mosaics, inflammatory punctuation, congestion, and ulceration) in a localized area in the transformation zone was found.

Two specimens containing exfoliated uterine cervical cells were collected from each patient using a cervical brush. One of these specimens was used to obtain a smear on a slide that was stained by the Papanicolaou method and analyzed by cytopathological examination, based on the Bethesda system [13]. We considered as normal cytology, or cytology without alterations, the samples that presented no pathological alteration or only inflammatory changes. Cytology with alterations were the samples in which we detected the presence of atypical squamous cells of undetermined significance (ASC-US), squamous intraepithelial lesions of low grade (LSIL) or squamous intraepithelial lesions of high-grade (HSIL).

The other cervical specimen was conditioned in a tube containing a preserving solution (PBS + vancomycin + nystatin) and sent to a laboratory where it was processed for DNA extraction.

### 2.3. Detection of HSV DNA

The tubes containing the cervical specimens were submitted to rigorous agitation before removal of the brush and were centrifuged at 300 ×g per 10 min. The supernatant was removed and the resulting pellet was processed for DNA extraction using QIAmp DNA Mini Kit (QIAGEN, Hilden, Germany) following the manufacturer's recommendations.

Around 30 ng of the DNA samples was submitted to a polymerase chain reaction (PCR) to amplify a 110 bp fragment of the human *β*-globin gene, using the primers PCO3+/PCO4+ [14] to analyze the quality of target DNA and the absence of PCR inhibitors. The products of PCR were submitted to electrophoresis on 8% polyacrylamide gel, followed by silver staining [15].

The positive samples for *β*-globin were analyzed for the presence of DNA from HSV-1 and HSV-2 in separated reactions by PCRs specific for each type. Each reaction was composed of a Master Mix (PROMEGA, Madison, WI, USA), 10 mM of each primer, and 2.5 mM of DNA sample, to a final volume of 25 *μ*L. The pair primers HSV-1a (5′-CCCTGTCTCGCGCGAGCCAC-3′) and HSV-1b (5′-TCAGCCACCCATACGCGTAA-3′), which amplify a fragment of 142 bp [16], were used to detect the presence of HSV-1, while the primers HSV-2 (A) (5′-GGACGAGGCGCCAAAGCACACG-3′) and HSV-2 (B) (5′-TCCGTCCAGTCGTTTATCTTCAC-3′), which generate a product 270 bp [17], were used to detect the presence of HSV-2. The conditions for both reactions were as follows: incubation at 50°C for 2 min and denaturation of DNA at 95°C for 5 min, followed by 40 cycles of 94°C for 1 min, 45 s at 58°C for annealing, an extension step at 72°C for 30 s, and a final extension step at 72°C for 10 min. The products of PCRs underwent vertical electrophoresis on polyacrylamide gel at 8% and subsequent visualization by silver staining [15].

### 2.4. Statistical Analysis

Statistical analysis of the results was performed using the chi-square test, while difference in proportion and associations between risk factors and HSV infection were analyzed by calculating the odds ratio (OR) and their respective confidence intervals (95% CI). Results were considered statistically significant at *P* value <0.05.

## 3. Results

The analysis of individual questionnaires revealed that the profile of the segment of the population studied was characterized by being composed of women aged 14–72 years, mean 30.3 ± 10.8 years. Most of them were less than 30 years of age, were of nonwhite ethnicity, were married or living in a stable relationship with her partner, had elementary education or less, had family income (monthly minimum wage) up to one minimum wage (approximately US$ 300.00), had their first sexual intercourse and the first pregnancy at age 18 or less, had more than one sexual partner in their lifetime, was not pregnant at the time of the survey, and had at least one pregnancy.

The presence of any of the serotypes of HSV alone or both together was detected in 95 women, revealing an overall prevalence of 40.3% of genital infection in the studied population (23.7% HSV-1 alone, 11.9% HSV-2 alone, and 4.7% the two serotypes together). The presence of at least one of the serotypes of HSV was detected in 36/106 (34.0%) pregnant women and in 59/130 (45.4%) nonpregnant women. We observed a significant higher prevalence rate of HSV-1 infection among non-pregnant women (30.0%), when compared to pregnant women (16.0%) (*P* = 0.0001). HSV-2 alone was found in 13/106 (12.3%) pregnant women and in 15/130 (11.5%) nonpregnant women. The coinfection by the two serotypes of HSV, in the same patient, was detected in 5.7% of pregnant and 4.7% of nonpregnant women (Table 1).

Most of the participants in this study underwent morphological examinations by colposcopy (92.8%) and cytology (96.6%) to detect the presence of changes of the uterine cervix. The colposcopic examination was carried out on 90/106 (84.9%) pregnant women and in 129/130 (99.2%) nonpregnant women. In the group of pregnant women, the colposcopic examination showed that 38/90 (42.2%) had visible abnormalities of the uterine cervix. Among nonpregnant women, the colposcopic examination showed that 45/129 (34.9%) had cervical changes (Table 2).

Cytological examination was performed on 100/106 (94.3%) pregnant women and in 128/130 (98.5%) nonpregnant women (Table 3). The cytological alterations were more prevalent in pregnant (41/100) than nonpregnant (22/128) women (*P* = 0.0001).

We also analyzed the correlation between the presence of genital infection by any of the two serotypes of HSV and morphological findings detected by colposcopy and cytological examinations. There was not any significant difference in the prevalence rates for HSV-1 between the groups with or without colposcopic abnormalities, in both pregnant and nonpregnant women. However, the prevalences of HSV-2 were significantly higher in women with colposcopy abnormalities in both pregnant and nonpregnant women, compared to those with normal colposcopy (Table 2). Similar results were found for women with or without cytological abnormalities (Table 3).

Analysis of the association between genital infection by HSV-1 and HSV-2, each individually, and the considered variables revealed the absence of association between genital infection with HSV-1 and all variables tested (Table 4). The genital tract infection by HSV-2 was found to be associated with morphological alterations detected by colposcopy or cytology, ethnicity, marital status, and number of lifetime sexual partners. The nonwhite and single women and those who had multiple partners presented a greater risk of acquiring genital HSV-2 infection (Table 5).

## 4. Discussion

It has been shown that the prevalence of genital tract infection by HSV-1 and HSV-2 varies substantially in the different geographic regions, including those within the same country [4]. In this study, we found an overall prevalence rate of 40.3%. We did not observe significant differences in prevalence rates by age distribution. The found prevalence rate was almost three times higher than that reported for United States women (14.0%) [4]. It was also higher than that described in a similar previous study involving women of Natal, Brazil (28.4%) [8].

Considering each serotype of the HSV individually, HSV-1 was more prevalent than HSV-2, in the segment of the population studied, presenting coherence with the results reported in other studies, such as those reported for women of the United States [18], for women of Israel [19], and for women of Natal [8]. This could be explained by changes in attitudes, behavior, and the sexual practices among adolescents, including oral-genital sexual contact. Furthermore, the use of condoms for vaginal intercourse could have reduced the exposure to genital infection by HSV-2, contributing to an increasing proportion of cases of HSV-1, compared with HSV-2 as cause of genital herpes infection [20].

HSV-1 was detected in 23.7% of women surveyed, presenting a prevalence rate almost equal to that found in a previous study for Natal residents women (23.0%) [8], but slightly smaller than the prevalence rate reported for women of the United States (32.0%) [4].

HSV-2 was detected in 11.9% of the study participants, representing double the prevalence rate of 5.4% reported by Pereira et al. in 2012 [8], but close to that described for American women (16.2%) [18]. Still the prevalence rate described in this study was very similar to that reported for women of Israel (13.3%) [21], while it was less than the estimated prevalence for women of South America [22] (varying between 20.0% and 40.0%). Coinfection by the two serotypes of HSV was detected in 4.7% of the women surveyed, which was above that in previous research conducted by Pereira et al. (2.3%) [8].

We found a higher prevalence rate of genital infection by HSV among nonpregnant women. HSV-1 was more prevalent among nonpregnant women, whereas HSV-2 presented similar prevalence among pregnant and nonpregnant women. The prevalence of genital HSV-1 infection found in the nonpregnant women was higher than that described for women of the United States (14.0%) [4]. Regarding pregnant women, HSV-1 was found with a prevalence rate very similar to that reported in a previous study in Natal's women (23.0%) [8].

The prevalence rate of HSV-2 was practically equal to that described for women in Korea [23] (17.0%) but was less than that reported for United States' women [3], approximately 22.0%. The prevalence rates found in nonpregnant women participating in this study for both HSV-1 and HSV-2 were higher than those described for women in the United States [4], which were 4.4% and 9.4%, respectively.

Pregnant women had higher percentages of abnormal results in both colposcopic and cytological examinations, when compared to nonpregnant women. These data suggest that pregnancy may be influencing the reactivation of HSV-2 infection.

We observed in this study a higher proportion of women infected with HSV-2 among those who had abnormal colposcopy and/or cytology, in both groups of pregnant and nonpregnant women. This suggests that HSV-2 has a higher ability to cause lesions in the genital tract. This result is consistent with the literature data reporting that the extent and frequency of symptomatic recurrent genital herpes is highly type-specific, with HSV-2 reactivations being much more frequent, outpacing HSV-1 by three to one [24]. Furthermore, it was shown that genital HSV-1 recurs infrequently and the rate of recurrences decreases rapidly over time, with a median recurrence rate declining by about 50% from the first to the second year of infection [25].

When we evaluated the existence or lack of association between genital HSV-1 infection and sociodemographic variables and sexual activity, it was found that there was no association between the variables tested and genital HSV-1 infection among women of this study, corroborating with the results obtained in a previous study conducted in Natal [8]. However, a significant association was observed between the variables of ethnicity, marital status, and number of sexual partners over a lifetime and the occurrence of genital infection by HSV-2. No association was observed with chronological age, education, and age at first sexual intercourse. These results are consistent with those obtained for Israel women [21], regarding education, age of first intercourse, and number of sexual partners. With regard to ethnicity, our data are also consistent with those described for United States' women [26].

This study presents some limitations. For example, the morphological alterations may not have been caused exclusively by HSV, once that there are other pathogens that cause cervical lesions, such as HPV, that were not analyzed in this study. Besides, the studied population is limited to women attended in two primary health care units and may not be representative of the female population of Natal. The analysis of a larger number of patients would allow obtaining more conclusive results.

In conclusion, results of the current study show a high prevalence rate of both HSV-1 and HSV-2 in the studied population. The significant proportion of pregnant women infected by HSV-2 that presented morphological alterations in the uterine cervix suggests the importance of including the colposcopy or cytology exams as part of routine prenatal care.

## Figures and Tables

**Table 1:** Prevalence of genital infection by herpes simplex virus types 1 and 2, individually or in coinfection, in pregnant and nonpregnant women.

PCR test for HSV	Condition			*P*
	Pregnant (%)	Nonpregnant (%)	Total	
Negative	70 (66.0)	71 (54.6)	141 (59.7)	
Positive	36 (34.0)	59 (45.4)	95 (40.3)	0.070
HSV-1	17 (16.0)	39 (30.0)	56 (23.7)	0.014*
HSV-2	13 (12.3)	15 (11.5)	28 (11.9)	0.755
HSV 1 + 2	6 (5.7)	5 (3.8)	11 (4.7)	0.754

Total	106 (100.0)	130 (100.0)		

*P*: *P* value using *χ* ^2^ test for comparison of HSV prevalence between pregnant and nonpregnant women.

*Statistically significant.

**Table 2:** Correlation between colposcopy examination and presence of herpes simplex virus types 1 and 2, single or in coinfection in pregnant and nonpregnant women.

Colposcopy	*N* (%)	PCR test positive for herpes simplex virus							
		HSV-1	%	HSV-2	%	HSV 1 + 2	%	Total	%
Pregnant (*N* = 106)								*P*1 = 0.263	
								*P*2 = 0.002*	
Without alterations	52 (49.1)	10	19.2	3	5.8	1	1.9	14	26.9
With alterations	38 (35.8)	7	18.4	8	21.1	5	13.2	20	52.6
Nonanalyzed	16 (15.1)	0	00.0	2	12.5	0	0.0	2	100.0
Nonpregnant (*N* = 130)								*P*1 = 0.520	
								*P*2 = 0.040*	
Without alterations	84 (64.6)	25	29.8	7	8.3	2	2.4	34	40.5
With alterations	45 (34.6)	14	31.1	8	17.4	3	6.7	25	54.3
Nonanalyzed	1 (0.8)	0	0.0	0	00.0	0	0.0	0	0.0

*P*1: *P* value using *χ* ^2^ test for correlation between the presence of genital infection by HSV-1 and morphological findings detected by colposcopy.

*P*2: *P* value using *χ* ^2^ test for correlation between the presence of genital infection by HSV-2 and morphological findings detected by colposcopy.

*Statistically significant.

**Table 3:** Correlation between cytological examination and presence of herpes simplex virus types 1 and 2, single or in coinfection in pregnant and nonpregnant women.

Cytology	*N* (%)	PCR test positive for herpes simplex virus							
		HSV-1	%	HSV-2	%	HSV 1 + 2	%	Total	%
Pregnant (*N* = 106)								*P*1 = 0.488	
								*P*2 = 0.001*	
Without alterations	59 (55.7)	10	16.9	3	5.1	1	1.7	14	23.7
With alterations	41 (38.7)	6	14.6	9	22.0	4	9.8	19	46.3
Nonanalyzed	06 (5.6)	1	16.7	1	16.7	1	16.7	3	50.0
Nonpregnant (*N* = 130)								*P*1 = 0.781	
								*P*2 = 0.022*	
Without alterations	106 (82.8)	34	32.1	10	9.4	3	2.8	47	44.3
With alterations	22 (17.2)	5	22.7	5	22.7	2	9.1	12	54.5
Nonanalyzed	2 (1.5)	0	0.0	0	0.0	0	0.0	0	0.0

*P*1: *P* value using *χ* ^2^ test for correlation between the presence of genital infection by HSV-1 and morphological findings detected by cytology.

*P*2: *P* value using *χ* ^2^ test for correlation between the presence of genital infection by HSV-2 and morphological findings detected by cytology.

*Statistically significant.

**Table 4:** Distribution of prevalence rates of HSV-1 infection, without or with HSV-2 according to variables considered and association of the odds ratios obtained by univariate logistic regression model.

Variable	Herpes simplex virus type 1						
	*N*	Positive	Negative	Pos (%)	OR	95% IC	*P*
Colposcopy							
With alterations	84	29	55	34.5	1.36	0.83–2.22	0.303
Without alterations	136	38	98	27.9		Reference	
Cytology							
With alterations	63	17	46	27.0	0.90	0.47–1.72	0.753
Without alterations	165	48	117	29.1		Reference	
Age (years)							
<30	119	37	82	31.1	2.08	0.68–6.79	0.163
31–45	89	25	64	28.1	1.80	0.56–6.08	0.279
>45	28	5	23	17.9	1.0	[Reference]	
Ethnicity							
White	106	29	77	27.4		[Reference]	
Nonwhite	130	38	92	29.2	1.10	0.60–2.02	0.751
Marital status							
Married or accompanied	182	54	128	29.7	1.33	0.63–2.85	0.423
Single	54	13	41	24.1		[Reference]	
Education							
Elementary or less	140	41	99	29.3	1.0	[Reference]	
High school	96	26	70	27.1	0.90	0.48–1.66	0.712
Age at first sexual intercourse							
<18	149	44	105	29.5	1.17	0.62–2.20	0.611
≥18	87	23	64	26.4		[Reference]	
Lifetime sex partners							
Multiple	142	41	101	28.9	1.06	0.57–1.98	0.840
Only one	94	26	68	27.6		[Reference]	

**Table 5:** Distribution of prevalence rates of HSV-2 infection, without or with HSV-1 according to variables considered and association of the odds ratios obtained by univariate logistic regression model.

Variable	Herpes simplex virus type 2						
	*N*	Positive	Negative	Pos (%)	OR	95% IC	*P*
Colposcopy							
With alterations	84	24	60	28.6	3.79	1.82–7.95	<0.001*
Without alterations	136	13	123	9.6	1	Reference	
Cytology							
With alterations	63	20	43	31.7	4.05	1.95–8.41	<0.001*
Without alterations	165	17	148	10.3	1	Reference	
Age (years)							
<30	119	19	100	16.0	1.14	0.32–4.38	0.826
31–45	89	16	73	18.0	1.32	0.36–5.18	0.651
>45	28	4	24	14.3		[Reference]	
Ethnicity							
White	106	11	91	10.4		[Reference]	
Nonwhite	130	28	106	21.5	2.19	0.98–4.97	0.038*
Marital status							
Single	54	14	40	25.9	2.20	0.98–4.89	0.034*
Married or accompanied	182	25	157	13.7		[Reference]	
Education							
Elementary or less	140	21	119	15.0		[Reference]	
High school	96	18	78	18.8	1.31	0.62–2.75	0.446
Age at first sexual intercourse							
<18	149	26	123	17.4	1.20	0.55–2.65	0.617
≥19	87	13	74	14.9		[Reference]	
Lifetime sex partners							
Multiple	142	30	112	21.1	2.53	1.08–6.08	0.019*
Only one	94	9	85	9.6		[Reference]	

*Statistically significant.
